# Exploring Children’s Digital Home Learning Environment: Cross-Cultural Construct Validation of the HLEQ

**DOI:** 10.3390/bs16071111

**Published:** 2026-07-03

**Authors:** Astrid Wirth, Edit Tóth, Ágnes Hódi

**Affiliations:** 1 Department of Education, University of Vienna, Sensengasse 3A, 1090 Vienna, Austria; 2 Department of Educational Evaluation and Planning, Institute of Education, University of Szeged, Egyetem u. 2, 6722 Szeged, Hungary; tothedit@edpsy.u-szeged.hu; 3 Department of Kindergarten Education, Institute of Applied Pedagogy, Juhász Gyula Faculty of Education, University of Szeged, Hattyas utca 10, 6725 Szeged, Hungary; hodi.agnes@szte.hu

**Keywords:** home learning environment, digital learning, early childhood, cross-cultural validation, exploratory factor analysis

## Abstract

Although digital media play an increasingly important role in young children’s home learning environment (HLE), validated tools that measure both traditional and digital aspects of the HLE are lacking. The present study examines the cross-cultural validity of the Greek Home Learning Environment Questionnaire (HLEQ) in Austrian and Hungarian contexts. A sample of *N* = 515 parents of preschool children (*M*_age_ = 62 months, *SD* = 13.2; 52% boys) was assessed. Confirmatory factor analyses supported the six-factor structure of the HLEQ across both countries, including digital learning activities as a distinct dimension. Reliability indices (Cronbach’s α, McDonald’s ω) indicated acceptable to good internal consistency across most scales. Multi-group analyses demonstrated configural and metric invariance, suggesting comparable factor structures and item–factor relations across countries. However, scalar invariance was not supported, indicating differences in item intercepts and limiting the comparability of latent mean levels. Descriptive findings revealed cross-national differences in most HLE dimensions. Overall, the results advance cross-cultural research on digital learning environments and support the structural validity of the HLEQ across cultural contexts while highlighting the importance of culturally sensitive interpretation when comparing HLE levels. Future research should examine how traditional and digital aspects of the HLE contribute to children’s development.

## 1. Introduction

The Home Learning Environment (HLE) encompasses the activities, interactions, and resources provided by parents that support children’s learning and development at home (Niklas et al., 2016; Sénéchal & LeFevre, 2002). Its importance for children’s cognitive, social, and socio-emotional development is well established, with longitudinal studies demonstrating lasting effects on academic achievement, literacy, numeracy, and broader competencies (e.g., Junge et al., 2021; Melhuish et al., 2008; Niklas & Schneider, 2017). These associations with developmental outcomes are shaped by the quality and context of interactions, while remaining independent of prior academic achievement and child and family characteristics (Lehrl et al., 2021; Niklas & Schneider, 2017).

Research on the HLE continues to face methodological and conceptual challenges (Wirth et al., 2023), particularly about capturing the HLE as a multidimensional construct that encompasses different interacting facets (Gregoriadis & Evangelou, 2022): One key challenge concerns the integration of digital media, which have become increasingly prevalent in children’s daily lives, into existing HLE frameworks in a theoretically meaningful way (Krousorati et al., 2022; Lehrl et al., 2021). Another challenge lies in the adequate assessment of the HLE itself, due to varying methodological approaches, which have been validated cross-culturally in only a few cases (e.g., Aminipour et al., 2020; Bradley et al., 1996). Cross-national comparisons are vital, because parental interactions with their children, and thereby the frequency and the meaning of HLE practices, are shaped by culturally based belief systems, comprising specific sets of values, policies and norms (Rubin & Chung, 2013). Specifically in regard to children’s use of digital media, parental mediation practices and societal attitudes toward children’s media use differ substantially across countries (OECD, 2025).

Therefore, the present study aims to address these gaps by examining the validity of the Greek Home Learning Environment Questionnaire (HLEQ; Krousorati et al., 2022) in Austrian and Hungarian samples, providing insights into both analogue and digital dimensions of the HLE across cultural contexts. The parent-reported measure HLEQ has been cross-validated in a Greek sample of parents of preschool-aged children. Exploratory and confirmatory factor analyses confirmed the multidimensional construct of the HLE by indicating six dimensions (Krousorati et al., 2022). By establishing cross-cultural validity, the present study contributes to determining whether observed differences in parental practices reflect true variation in families’ learning environments or merely differences in how constructs are measured and interpreted across countries.

### 1.1. Children’s Digital Media Use in Hungary and Austria

In recent years, children’s exposure to digital media has increased substantially; however, this development is accompanied by considerable variability across countries and cultural contexts (Chaudron et al., 2018; OECD, 2025). Importantly, most large-scale data on media use focus on school-aged children or adolescents, whereas comparatively little is known about media use in the preschool years (OECD, 2025). Available international findings from Australia, Germany, and the USA indicate that children grow up in highly digitalized households, where media use in early childhood is often dominated by passive forms of engagement, particularly video viewing, while interactive uses such as gaming or digital reading play a comparatively smaller role (e.g., Common Sense Media, 2025; Huber et al., 2018; Medienpädagogischer Forschungsverbund Südwest, 2023).

Data from Austria and Hungary indicate some differences in children’s media use between the two countries. While digital devices are widely available in both countries, levels of engagement differ: In Hungary, digital media use appears to be highly prevalent, with approximately 81% of three-year-olds already using digital devices, increasing to 93% by the age of six (Szőke-Milinte, 2025). In Austria, levels of engagement are slightly lower, with around 72% of children aged 0 to 6 using digital media at least occasionally, rising to 81% among 3- to 6-year-olds (Kaiser-Müller, 2020).

A more pronounced divergence emerges with respect to device preferences: In Hungary, television or smart TV is the dominant medium, used by 85% of preschool children and serving as the primary device for 70%. Smartphones represent the second most common device (47%), followed by tablets (22%), computers or laptops (11%), and game consoles (8%) (Szőke-Milinte, 2025). In Austria, children most frequently use tablets (32%), followed by smartphones (30%) and internet-enabled TVs (21%), while only 4% use computers or laptops (Kaiser-Müller, 2020). In addition, personal device ownership is less common in Hungary (11%) compared to Austria (22%) (Saferinternet.at, 2020; Szőke-Milinte, 2025).

These differences in access and device use point to broader variations in how digital media are integrated into family contexts. In this regard, digital media use within the home is influenced by several factors, including children’s age and gender (Kucirkova et al., 2018) as well as socioeconomic background (Nikken & Opree, 2018; Vandewater et al., 2007).

In Hungary, older preschool children tend to use digital devices more frequently than younger ones, while gender differences are generally small, with the exception of gaming, which is almost twice as common among boys (11%) as among girls (6%) (Szőke-Milinte, 2025). Socioeconomic differences are also evident, with higher overall use among children of younger and/or less-educated parents. Device preferences vary accordingly, with higher smartphone use in lower-educated families, whereas television or smart TV is more often the primary device in higher-educated households (Szőke-Milinte, 2025). Comparable detailed data for Austria are not available.

Taken together, these cross-national differences are reflected not only in the frequency of media use but also in how digital media are embedded in children’s everyday lives and family practices, raising important questions about how these experiences interact with and shape the HLE.

### 1.2. The Transformation of the HLE in the Digital Age

The increasing integration of digital media into children’s everyday lives necessitates a reconsideration of the HLE. Traditionally, the HLE has been conceptualized in terms of analogue resources and activities, such as shared book reading, playing board games and other practices supporting children’s learning and development (Niklas et al., 2016; Sénéchal & LeFevre, 2002). With the growing prevalence of digital media in family contexts, it becomes relevant to examine how digital practices contribute to or reshape children’s learning environments.

The impact of digital media on children’s learning and development depends strongly on usage patterns. A growing body of research, including meta-analyses, indicates that targeted use of digital tools, particularly learning apps, can support children’s competency development across various areas (Griffith et al., 2020; Kim et al., 2021). However, such effects are primarily observed in structured or guided contexts, often within experimental or quasi-experimental designs (Herodotou, 2018). In contrast, digital media use in everyday family settings is more heterogeneous and less structured, which may limit its developmental benefits or even signal potential risks associated with certain patterns of media use (e.g., Paulus et al., 2021; Schwarzer et al., 2022). Moreover, high levels of screen time have been associated with lower parental efficacy and fewer literacy-related activities in the home, particularly in relation to frequent television or tablet use (Chen et al., 2020). Importantly, not only the amount but also the kind of digital interaction appears to be crucial, including distinctions between passive activities (e.g., watching videos or television) and more active or educational uses (e.g., learning apps), as well as parental mediation practices. Consistent with the high prevalence of television use in Hungary, which may indicate more passive forms of engagement, parents most frequently report entertainment and play as the primary reasons for their children’s digital media use (77%), followed by learning or developmental purposes (51%). Additional motivations include keeping the child occupied (18%), lack of parental time or energy (12%), and calming the child (5%) (Szőke-Milinte, 2025). Although comparable data on parental motivations are not available for Austria, reported media activities—such as watching videos (73%), viewing photos (61%), listening to music (61%), and playing games (51%)—also point to predominantly entertainment-oriented use (Kaiser-Müller, 2020). At the same time, there is evidence of a developmental shift toward more educational uses as children approach school entry (Eichen et al., 2021).

Parental mediation and media-related parenting practices appear to be important in both countries. However, families in Hungary report substantially higher levels of restrictive mediation, both in terms of content restrictions (81% vs. 42%) and time limits (81% vs. 44%), compared to Austria. Approximately half of parents in both countries report being present during their children’s media use (46% in Hungary, 57% in Austria). These findings contrast with commonly reported patterns in the literature, where parents often indicate allowing children to use digital media to manage daily demands, such as completing household tasks (e.g., Eichen et al., 2021) and may reflect differences between reported attitudes and actual practices.

These findings raise conceptual questions regarding the role of digital media within the HLE, and whether digital media use should be integrated into existing HLE frameworks or considered as a distinct dimension. In accordance with the analogue HLE, children’s digital HLE can be characterized by access to digital tools, frequency of use, and the quality of support provided during their use (Lehrl et al., 2021). Some authors argue for distinguishing between analog and digital home learning environments due to their potentially different associations with children’s outcomes: While analog literacy practices are consistently linked to positive developmental effects, findings regarding digital media use are more heterogeneous (Jáñez et al., 2024; Segers & Kleemans, 2020). In contrast, Krousorati et al. (2022) have incorporated digital activities such as reading e-books, using learning apps, browsing the internet, and playing video games into their definition of the HLE to gain broader insights into the quality of contemporary learning environments. This view is supported by Sonnenschein et al. (2021), describing literacy and digital activities as two significantly correlated aspects of the HLE.

Overall, current findings point to the need for a more differentiated conceptualization of the HLE that accounts for variation in digital practices. Although digital media are increasingly relevant in early childhood, evidence on how they are embedded in everyday family contexts and how they interact with established components of the HLE remains limited. Taken together, current studies highlight that the HLE is evolving in the digital age, requiring theoretical and methodological approaches that better capture the interrelation of traditional and digital learning environments across diverse cultural contexts.

### 1.3. The Present Study

Not only are digital activities shaped by cultural context and family background, but this also applies to the HLE itself. While associations between the HLE, families’ socioeconomic background, and children’s developmental outcomes appear to be relatively consistent across many cultural contexts, differences in the structure and composition of the HLE can be observed even among Western societies (Bradley et al., 1996). From a broader cross-cultural perspective, these differences are particularly pronounced for dimensions related to socio-emotional support, but also extend to the emphasis placed on learning-oriented activities, such as shared reading and teaching letters, versus more play-based activities, including arts and creative engagement (Aminipour et al., 2020; Bradley et al., 1996). However, despite these cross-cultural variations, many instruments used to assess the HLE have not been systematically validated across different cultural contexts.

Therefore, the present study aims to examine the HLE from a cross-cultural perspective, with a particular focus on digital activities. Specifically, it seeks to validate the Greek Home Learning Environment Questionnaire (HLEQ) in Austrian and Hungarian contexts and to investigate potential cultural differences in both digital and broader HLE dimensions. To this end, the study pursues two main objectives: (a) to assess the cross-cultural validity of the HLEQ as a measure of the (digital) HLE, and (b) to compare the characteristics of digital and other HLE dimensions between the two countries. Accordingly, the study addresses the following research questions:

- RQ1: What are the characteristics of the different HLE dimensions, and are there significant differences in their levels between Austria and Hungary? Given existing evidence on cross-cultural variation in HLE practices, differences in the levels of specific HLE dimensions between Austria and Hungary are expected (Aminipour et al., 2020; Bradley et al., 1996).
- RQ2: Do levels of the different HLE dimensions vary as a function of children’s age and gender? Previous research suggests that patterns of digital media use differ depending on child characteristics. For example, representative data from Germany indicate gender differences in digital media use, such as television viewing, even among children under the age of five (Medienpädagogischer Forschungsverbund Südwest, 2023). In addition, studies have found gender-specific usage patterns for digital applications and video games in early childhood (Schiele et al., 2025; Szőke-Milinte, 2025). Based on this evidence, we expect differences in the digital dimension of the HLE depending on children’s age and gender.
- RQ3: Does the factor structure of the HLEQ replicate in samples from Austria and Hungary, and is the measurement model comparable across both countries? Following Krousorati et al. (2022), we would expect a six-factor structure, with digital learning activities emerging as a distinct factor.

## 2. Materials and Methods

### 2.1. Sample and Procedure

Our sample consisted of *N* = 515 parents of preschool children attending kindergarten, with *n* = 339 parents from Hungary and *n* = 176 parents from Austria. In *n* = 410 cases, the child’s mother completed the questionnaire, in *n* = 33 cases the father, and in *n* = 69 cases both parents jointly.

The children were on average *M* = 62 months old (*SD* = 13.2, range = 25–93 months), and gender was approximately equally distributed, with 52% boys. There were no significant gender differences between the country subsamples. However, children’s age differed significantly, with children from Hungary being significantly older than those from Austria (*M*_HUN_ = 63.73, *SD* = 12.54; *M*_AT_ = 57.64, *SD* = 13.12; *t*(513) = 4.70, *p* < 0.001).

In the Hungarian sample, all participating parents reported speaking exclusively or predominantly Hungarian at home. In the Austrian sample, 13.4% of parents (*n* = 24) reported a migration background, defined as speaking a language other than German at home equally often or more frequently than German.

Regarding parental education, in the Hungarian sample, *n* = 136 (40.1%) of families reported that both parents had obtained at least a high school diploma. In the Austrian sample, this proportion was *n* = 148 (83.2%). A detailed summary table containing demographic data for both samples can be found in Supplementary Table S1.

Most parents were recruited through their children’s kindergartens (*n* = 394). Additional participants were recruited via social media parent groups (*n* = 122). Parents were invited to participate by completing the HLEQ along with additional background questions. Participation was possible either via paper-and-pencil questionnaires or through an online survey.

Prior to participation, parents received detailed study information in accessible language and provided written informed consent. The study was approved by the ethics committee of the University of Vienna, Austria (reference number: 01345).

### 2.2. Measures

Children’s HLE was assessed using the written questionnaire HLEQ (Krousorati et al., 2022). The HLEQ is a self-report instrument comprising 32 items that assess activities and interactions across six dimensions of the HLE: The first scale, *Indoor learning activities*, comprises six items on shared reading and writing, crafting and different kinds of indoor games. An example item is: “Plays role-play games with the child (e.g., the baker and the customer)”. The second scale, *Digital learning activities* (five items), focuses on diverse digital activities, such as the use of e-books, literacy/numeracy learning apps, internet and video games. An example item is: “Surfs the web with the child (e.g., to find a story or a song)”. The third scale, named *Warmth/Support* (six items), captures emotionally supportive parental behaviors during shared activities and reflects the extent to which parents respond sensitively to their child’s needs, encourage persistence, and foster a positive learning climate. An example item is: “Encourages the child to keep up with an activity when the child finds it challenging”. The fourth scale, *Outdoor learning activities,* consists of five items assessing parents’ engagement in cultural, artistic, and physical activities outside the home environment. An example item is: “Takes the child to cultural events (e.g., cinema, theatre, museums)”. The fifth scale, *Conflict*, includes five items measuring negative and conflictual interactions that may occur during shared activities, reflecting parental frustration, irritability, and critical responses toward the child. An example item is: “Gets upset and raises his/her voice when the child does not meet his/her expectations in any activity”. The sixth scale, *Inconsistent discipline*, comprises five items assessing the extent to which parents apply rules and consequences inconsistently. An example item is: “Gives in to the child’s demands when the child throws a tantrum (e.g., screams for sweets in a supermarket)”. All items were rated on 6-point Likert scales ranging from 1 (never) to 6 (always). The instrument was originally developed and validated in the Greek educational context.

For the present study, the originally published English version (Krousorati et al., 2022) was translated into German and Hungarian. In a qualitative pilot step, the translated versions were reviewed by a small sample of parents in both countries to ensure clarity, comprehensibility, and cultural appropriateness prior to their use in the main quantitative study.

All research materials, including the German and Hungarian versions of the HLEQ as well as the fully anonymized dataset, are publicly available at https://osf.io/7syaz (accessed on 16 June 2026).

### 2.3. Analytic Approach

Descriptive statistics (means, standard deviations, and scale ranges) as well as bivariate correlations among all study variables and covariance analyses (ANCOVA) were conducted using IBM SPSS Statistics Version 31 (IBM Corp., 2024). An ANCOVA was conducted to examine differences in HLE dimensions between Austria and Hungary, in regard to children’s age, parental education, as well as gender and family language (Research question 1).

To examine age- and gender-related differences in HLE dimensions, an additional ANCOVA model was estimated including gender as a fixed factor, child age (in months) as a covariate, and their interaction term (Gender × Age), while country, family language, and parental education were retained as control variables (Research question 2).

A confirmatory factor analysis (CFA) with six latent factors, representing the six dimensions of the HLE, was conducted to examine the factor structure of the HLEQ (Research question 3). The analysis was performed in R (R Studio Team, 2024) using the lavaan package (version 0.6-21). We applied maximum likelihood estimation with robust standard errors (MLR) to account for potential non-normality in the data. Based on modification indices, we added residual covariances between items within the same subscale for one to three items across five subscales, where theoretically justified.

Model fit was evaluated using multiple indices, following conventional reporting guidelines for structural equation modeling (Kline, 2023). Following recommendations by Hu and Bentler (1999), values of CFI and TLI ≥ 0.95, RMSEA ≤ 0.06, and SRMR ≤ 0.08 were considered indicative of good model fit.

## 3. Results

### 3.1. Descriptive Statistics and Correlational Analyses

Table 1 presents the descriptive statistics and reliability coefficients (Means, Standard Deviations, Scale ranges, McDonald’s Omega) for all HLEQ dimensions separately for Hungary and Austria. Overall, mean scores were comparable across countries, with slightly higher values in the Austrian sample for all dimensions. Internal consistency was acceptable to good for most scales in both samples. However, somewhat lower reliability coefficients were observed for the scales *Indoor learning activities* and *Outdoor learning activities*, particularly in the Austrian sample.

Table 2 presents the correlations among the HLEQ dimensions as well as child and family characteristics, reported separately for Hungary and Austria.

Overall, the pattern of correlations was largely comparable across both countries. The positive dimensions of the HLE (*Indoor learning activities*, *Warmth/Support*, and *Outdoor learning activities*) were moderately to strongly intercorrelated in both samples (*r* = 0.24–0.48). In contrast, the negative dimensions (*Conflict* and *Inconsistent discipline*) were strongly positively associated (r = 0.43–0.64) and showed negative correlations with the positive HLE dimensions.

Digital learning activities showed a particularly distinct pattern. In the Austrian sample, they were positively associated with all other HLE dimensions, while in the Hungarian sample, associations were generally weaker and only slightly significant for the dimension Warmth/Support. With regard to background variables, child age was positively correlated with digital learning activities in both countries, suggesting increased engagement with digital media as children grow older. Family education was negatively related to digital learning activities in both countries, and no associations between digital learning activities and children’s gender were found.

### 3.2. Cross-Cultural Differences in the HLE

To answer Research question 1, an analysis of covariance (ANCOVA) was conducted to examine differences in HLE dimensions between both countries. The six dimensions of the HLE were included as dependent variables. Child age (in months), maternal education, and paternal education were entered as covariates, while gender and family language were included as categorical factors. All assumptions of ANCOVA were tested prior to analysis. The assumption of homogeneity of regression slopes was met, as none of the interactions between countries and the covariates were statistically significant (*p*s > 0.05). Levene’s test indicated that the assumption of homogeneity of variances was not violated.

Across both countries, the dimensions of the HLE showed distinct patterns in their adjusted mean levels. *Indoor learning activities* showed the highest values, with means exceeding 5 in both Austria (*M* = 5.12) and Hungary (*M* = 5.21). *Digital learning activities* were reported at moderately high levels, with means ranging from 3.94 in Hungary to 4.38 in Austria, followed by *Outdoor learning activities*, which ranged from 3.15 to 3.49. In contrast, *Warmth/Support* was characterized by lower mean levels, varying between 1.68 (Hungary) and 2.17 (Austria). The dimensions *Conflict* and *Inconsistent discipline* also fell within a lower range overall, with means spanning from 1.67 to 2.65 across countries. Taken together, the adjusted means indicate a clear differentiation across dimensions, with higher levels observed for learning-related activities and comparatively lower levels for the remaining dimensions (see Table 3).

Estimated marginal means indicated that, after controlling for covariates, children in Austria scored higher than those in Hungary across all dimensions where significant group differences were observed, with effect sizes ranging from small to moderate (see Table 3). No significant interactions between country and the covariates were found.

With regard to Research question 2, an additional ANCOVA model was conducted to examine age- and gender-related differences in the level of the HLE dimensions, controlling for country, family language, and parental education. Significant main effects of child age (in months) were observed for *Indoor learning activities*, *F*(1, 485) = 6.38, *p* < 0.05, η^2^_p_ = 0.013, *Digital learning activities*, *F*(1, 485) = 17.50, *p* < 0.001, η^2^_p_ = 0.035, and *Outdoor learning activities*, *F*(1, 485) = 6.32, *p* < 0.05, η^2^_p_ = 0.013, indicating age-related variation in these dimensions. In all cases, the direction of the effects indicated that older children experienced higher levels of stimulation in these domains. No significant age effects were found for *Warmth/Support, Conflict*, or *Inconsistent discipline*. A significant main effect of gender was observed only for *Indoor learning activities*, *F*(1, 485) = 7.89, *p* < 0.01, η^2^_p_ = 0.016, with girls showing higher levels than boys, whereas no significant gender effects were found for the remaining dimensions. In addition, a significant Gender × Age interaction was found for *Indoor learning activities*, *F*(1, 485) = 8.03, *p* < 0.01, η^2^_p_ = 0.016, indicating that age-related increases differed between boys and girls, while no significant interaction effects were observed for the other dimensions. Results indicated a small age-related decline among girls, whereas levels remained relatively stable among boys.

#### 3.2.1. Confirmatory Factor Analyses

Concerning Research question 3, confirmatory factor analyses (CFA) were conducted within a multi-group framework using robust maximum likelihood estimation (MLR) to examine whether the six-factor structure established in the original Greek validation study could be replicated in the Austrian and Hungarian samples,. The specified model comprised six latent factors: *Indoor Activities* (IA), *Digital learning activities* (DA), *Warmth/Support* (WS), *Outdoor learning activities* (OA), *Conflict* (CO), and *Inconsistent discipline* (ID). Based on modification indices and theoretical considerations, several residual covariances between items within the same subscales were included to account for local item dependencies. Specifically, residual correlations were added between six item pairs on the scales OA, DA, WS, and CO (see Supplementary Table S2). All added residual correlations were within the same factor and therefore did not compromise the conceptual distinctiveness of the latent constructs.

The configural model showed acceptable fit to the data, with robust fit indices of CFI = 0.905, RMSEA = 0.047, and SRMR = 0.064. Although the chi-square test was significant, this was expected given the sample size and the complexity of the model. Overall, the fit indices indicate that the six-factor structure provides an adequate representation of the data in both national samples.

All factor loadings were statistically significant (*p* < 0.001) in both the Austrian and Hungarian samples. The proportion of variance explained by the latent factors (*R*^2^) ranged from 0.11 to 0.68 in Hungary and from 0.15 to 0.69 in Austria, indicating moderate to strong explanatory power across most items. Standardized factor loadings ranged from λ = 0.32 to 0.83 in Hungary and from λ = 0.39 to 0.83 in Austria (see Supplementary Table S3). Overall, items showed moderate to strong associations with their intended latent constructs, with particularly high loadings for *Warmth/Support*, *Conflict*, and *Inconsistent discipline* (λ ≥ 0.70), indicating robust measurement of these dimensions across both countries. In contrast, several items of the *Indoor learning activities* and *Outdoor learning activities* scales exhibited comparatively lower loadings (λ ≈ 0.32–0.43), especially in the Hungarian sample, suggesting weaker associations with the underlying constructs. Despite this variability, the overall loading pattern was largely comparable across Austria and Hungary, supporting the cross-cultural stability of the six-factor structure. Residual correlations were consistently observed within subscales and supported the presence of shared item-specific variance beyond the latent factors.

Taken together, these findings support the structural validity of the six-factor HLEQ model in both the Austrian and Hungarian samples.

#### 3.2.2. Measurement Invariance Across Austria and Hungary

Measurement invariance was examined using multi-group CFA following a stepwise procedure, testing configural, metric, scalar, and exploratory partial scalar invariance, as shown in Table 4. Model comparisons were primarily evaluated based on changes in robust CFI (ΔCFI), with values ≤ 0.01 indicating invariance. The configural invariance model demonstrated acceptable fit (robust CFI = 0.905, RMSEA = 0.047, SRMR = 0.064), indicating that the six-factor structure was comparable across Austria and Hungary.

Constraining factor loadings to equality across groups resulted in only a negligible decrease in model fit (robust CFI = 0.901, RMSEA = 0.048, SRMR = 0.069, ΔCFI = 0.004), supporting metric invariance. This suggests that the items relate to their respective latent constructs in a similar way across both countries. In contrast, imposing equality constraints on both factor loadings and item intercepts led to a substantial deterioration in model fit (robust CFI = 0.836, RMSEA = 0.061, SRMR = 0.078, ΔCFI = 0.065), indicating that scalar invariance was not supported. This suggests that item intercepts differ between the Austrian and Hungarian samples.

To further explore potential sources of non-invariance, an exploratory partial scalar invariance model was estimated in which the intercepts of four items (see Supplementary Table S4) were freely estimated across groups. This resulted in a modest improvement in model fit (robust CFI = 0.863, RMSEA = 0.056, SRMR = 0.074). However, model fit remained notably worse than in the metric invariance model (ΔCFI = 0.038), indicating that partial scalar invariance could not be established convincingly.

Overall, these results indicate that while the HLEQ demonstrates configural and metric invariance across Austria and Hungary, scalar invariance is not supported. Consequently, comparisons of associations between constructs across countries are justified, whereas comparisons of latent mean levels should be interpreted with caution.

## 4. Discussion

The present study examined the cross-cultural applicability of the Home Learning Environment Questionnaire (HLEQ; Krousorati et al., 2022) in Austrian and Hungarian samples, focusing on the role of digital learning activities within the broader HLE. More specifically, the study addressed three questions: (1) whether levels of HLE dimensions differed between Austria and Hungary, (2) whether these dimensions varied as a function of children’s age and gender, and (3) whether the HLEQ factor structure could be replicated across both countries. The findings support the six-factor structure of the original instrument and show that it can be applied not only in Greece but also in two Central European countries, Austria and Hungary. At the same time, the study reveals meaningful cross-cultural differences in several HLE dimensions. These results add to research highlighting the importance of multidimensional conceptualizations of the HLE (e.g., Gregoriadis & Evangelou, 2022; Niklas et al., 2016), while addressing the lack of cross-culturally validated instruments (Aminipour et al., 2020; Bradley et al., 1996).

A central finding concerns measurement invariance across Austria and Hungary. The HLEQ showed configural and metric invariance across Austria and Hungary, suggesting that the same latent constructs are captured and that items relate similarly to their underlying factors in both groups. This indicates that core dimensions of the HLE, such as analogue and digital learning activities as well as emotional support and warmth, can be meaningfully compared at the structural level across cultural contexts. However, scalar invariance was not supported, and partial scalar invariance could not be established. This suggests that item intercepts differ between countries and that similar observed scores do not necessarily reflect equivalent latent levels. This is consistent with previous research showing that while the overall structure of the HLE may be relatively stable, its expression is shaped by cultural norms, parenting beliefs, and contextual conditions (Aminipour et al., 2020; Bradley et al., 1996; Carollo et al., 2025). Specifically in regard to digital media use in early childhood, cultural norms determine how digital technologies are used and what level of use is considered acceptable or inappropriate (ÖIAT, 2026). Therefore, the lack of scalar invariance is not necessarily a methodological limitation but may show meaningful cultural differences in how parent–child interactions reflected in different HLE-dimensions are expressed. The factor loadings also support this interpretation: Across both countries, all items loaded significantly on their intended factors. Standardized loadings ranged from λ = 0.32 to 0.83 in Hungary and from λ = 0.39 to 0.83 in Austria, while explained variance ranged from *R*^2^ = 0.11 to 0.68 and *R*^2^ = 0.15 to 0.69, respectively. Particularly strong loadings were observed for *Warmth/Support*, *Conflict*, and *Inconsistent Discipline*, suggesting that these HLE dimensions focusing on educational and socio-emotional interactions between parents and children may be captured relatively consistently across cultural contexts. Interestingly, in the present sample, these dimensions of the HLE also remained stable across children’s ages, and prior research showed particularly strong links between these socio-emotional aspects of the HLE and children’s developmental outcomes (Junge et al., 2021; Melhuish et al., 2008). Weaker loadings for *Indoor learning activities* and especially *Outdoor learning activities*, particularly in the Hungarian sample, suggest that activity-based dimensions may be more sensitive to contextual variation. While stimulating home environments are generally associated with positive cognitive and language development (Lobo et al., 2025; Niklas & Schneider, 2017), the specific composition of such environments can vary considerably across cultural and socioeconomic contexts (Aminipour et al., 2020; Bradley et al., 1996).

In addition, the study also assessed cross-national differences in HLE dimensions. After controlling for relevant covariates, Austrian families reported higher levels of *Digital learning activities, Warmth/support, Outdoor learning activities, Conflict*, and *Inconsistent discipline* than Hungarian families, whereas no significant differences were found for *Indoor learning activities*. The absence of differences in *Indoor learning activities* may reflect relatively common early childhood practices, such as shared reading and playing, which have consistently been identified as central components of the HLE (Niklas & Schneider, 2017; Sénéchal & LeFevre, 2002), whereas variations in the remaining dimensions may imply broader patterns of interaction and regulation within families according to contextual and cultural factors. A comparison with the distributions in the original Greek sample reveals yet another differentiated picture (Krousorati et al., 2022): Although the individual item on shared book reading on the *Indoor learning activities* dimension was similarly pronounced, the dimensions *Indoor* and *Digital learning activities* were generally less pronounced than in Hungary and Austria. Additionally, parents in the Greek sample reported similar levels of *Outdoor learning activities* and *Conflict* to parents in Hungary and Austria; however, the dimension of Warmth/support was noticeably more pronounced than in Hungary and Austria.

These cross-national differences are particularly interesting with regard to the digital dimension of the HLE. In comparison with international evidence proving substantial variability in the availability of digital technologies for preschoolers (Chaudron et al., 2018; OECD, 2025), the present findings in our sample suggest that differences emerge not only in the prevalence but also in the integration of digital activities within family routines. In the Austrian sample, digital learning activities were more strongly associated with other HLE dimensions, suggesting a closer embedding in broader learning-oriented practices. Families who engage more frequently in traditional home learning activities also appear to integrate digital media into these practices more systematically. In contrast, the weaker associations observed in the Hungarian sample between digital learning activities and other home learning practices may indicate that digital media occupy a more independent role within family life and are less closely embedded in broader educational interactions. This interpretation is consistent with previous evidence suggesting that digital media use in Hungarian families, such as gaming, is primarily driven by entertainment-oriented purposes (Korpics et al., 2026).

The findings of this study therefore contribute to the ongoing debate on whether digital activities should be conceptualized as an integral component of the global HLE or as a distinct domain (Bonanati et al., 2022; Krousorati et al., 2022; Sonnenschein et al., 2021). Our results suggest that this may be a culture-dependent question, as patterns of media use are possibly shaped by culturally embedded practices and historically developed differences in digital socialization.

However, more pragmatic explanations may also be considered. Given that Hungary can be described as a “small market” with comparatively complex local market conditions in relation to the global diffusion of digital technologies and service providers (Csordás & Gálik, 2023), it is plausible that the availability and diversity of high-quality digital learning resources for preschool-aged children is more limited than in the German-speaking Austrian context. This could further contribute to differences in how digital media are integrated into early learning environments within families.

The analyses of age and gender differences further highlight the differentiated nature of the HLE. Children’s age was positively associated with *Indoor, Digital, and Outdoor learning activities*, indicating that older children experience higher levels of engagement in these domains. Developmental research shows that engagement in both analogue and digital learning activities increases with age as children gain autonomy and access to resources, specifically digital tools (Eichen et al., 2021; Kucirkova et al., 2018; Szőke-Milinte, 2025), a pattern that is also evident in the present findings. In contrast, relational dimensions such as *Warmth/support, Conflict, and Inconsistent discipline* were not significantly related to age, suggesting relative stability in parents’ educational practices during early childhood. Findings revealed a significant Gender × Age interaction indicating diverging directions for genders across ages. Analyses showed that the frequency of indoor learning activities remained relatively stable across age among boys, whereas a slight decline was observed among girls. Although the effect size was small, this finding suggests that developmental changes in parent–child engagement in shared indoor learning activities may differ by gender. However, given the cross-sectional design of the study, these age-related patterns should not be interpreted as developmental trajectories. These findings reflect previous research on gender-specific patterns in early learning and media use (Medienpädagogischer Forschungsverbund Südwest, 2023; Schiele et al., 2025).

All in all, the present findings contribute to a more differentiated understanding of the HLE in the digital age and across cultures. Although structural relations between HLE dimensions appear similar, differences in response patterns likely reflect culturally specific norms and expectations.

### 4.1. Limitations

The present study has several limitations that should be considered. First, it relied on parental self-report surveys, which may be subject to social desirability bias and cross-cultural differences in reporting styles. This limitation is well documented in HLE research, where different methodological approaches, including observational measures, can provide complementary insights (Bradley & Caldwell, 1984; Lehrl et al., 2021; Linver et al., 2004). Second, the Austrian and Hungarian samples differed in several background characteristics, including parental education, child age, and sample size. Although these factors were statistically controlled where appropriate, they may nevertheless have influenced the results. In particular, the substantially larger Hungarian sample may have led to greater statistical power and more precise parameter estimates than the Austrian sample. While both samples were sufficiently large for the conducted CFA and measurement invariance analyses, the imbalance could have affected the stability of country-specific estimates. With regard to educational differences in both samples, it should be noted that the tertiary education rate in Austria is generally higher than in Hungary (European Commission, 2025). However, both samples are better educated than the national average for their respective countries, particularly the Austrian sample. Although Hungarian children were on average older than Austrian children, all country comparisons statistically controlled for child age. Thus, the observed cross-national differences cannot be explained by the age distribution of the samples alone. Third, participants were recruited through both kindergartens and social media parent groups. Exploratory analyses indicated that parents recruited via social media reported higher levels of digital learning activities than those recruited through kindergartens, although the groups did not differ with regard to parental education. Consequently, the recruitment strategy may have influenced the observed levels of digital learning activities, and the sample may not be fully representative of the broader population of families with preschool children.

### 4.2. Future Directions

Future research should further investigate the cross-cultural functioning of HLE measures, including potential adaptations to improve scalar equivalence. Conducting latent profile analyses to identify different HLE profiles as well as combining self-report data with observational approaches could also provide a more comprehensive understanding of how both traditional and digital HLE dimensions unfold in children’s daily lives (Krousorati et al., 2022; Wirth et al., 2023).

The present findings support the inclusion of digital learning activities as a distinct dimension, yet it remains unclear whether digital practices should be conceptualized as an additional component of the traditional HLE or whether increasing digitalization requires a broader reconceptualization of the HLE itself. Future research should therefore move beyond asking whether digital media belong to the HLE and instead investigate how different forms of digital engagement shape children’s learning environments (Bonanati et al., 2022). This includes examining different types of media, active/creative or passive digital media use, parental mediation strategies, and parent–child interactions during digital media use, as well as to how these practices are embedded in everyday family learning environments and may vary across cultural contexts.

Further, recent research suggests that the developmental effects of digital media depend less on mere access than on the quality and context of digital interactions, including parental mediation and shared engagement (Harverson et al., 2025; Lehrl et al., 2021; Segers & Kleemans, 2020). The validated HLEQ therefore offers new opportunities to examine which patterns of digital and traditional learning activities are most strongly associated with children’s cognitive, linguistic, socio-emotional, and early academic development. Such evidence could inform interventions and educational policies aimed at fostering high-quality home learning environments.

## 5. Conclusions

The present study shows that the HLE is both structurally coherent across different European countries and culturally embedded. The validated parent-report measure HLEQ demonstrated a stable six-factor structure and metric invariance across Austria and Hungary, indicating that core dimensions of the HLE are comparable across contexts. However, the lack of scalar invariance suggests that mean-level comparisons should be interpreted with caution, as the expression of HLE dimensions differs across cultural settings. Substantively, the findings highlight meaningful cross-national variation, as Austrian families reported higher levels than Hungarian families in all HLE dimensions except *Indoor learning activities*, which did not differ between countries. Particularly in regard to digital learning activities, the integration into broader home learning practices varied between both countries, raising important questions about the origins of these cultural differences in digital media use within families.

Overall, the study contributes to a more nuanced understanding of the HLE in the digital age and underscores the importance of culturally sensitive measurement when comparing home learning environments across countries.

## Figures and Tables

**Table 1:** Descriptive statistics of the HLEQ dimensions in Hungary and Austria.

	Hungary			Austria		
HLEQ Dimensions	*M* (*SD*)	Range	*ω*	*M* (*SD*)	Range	*ω*
Indoor learning activities	4.07 (0.78)	1–6	0.74	4.52 (0.72)	2–6	0.64
Digital learning activities	1.86 (0.75)	1–4	0.75	2.09 (0.77)	1–4	0.75
Warmth/Support	5.09 (0.68)	3–6	0.85	5.13 (0.65)	3–6	0.81
Outdoor learning activities	3.18 (0.90)	1–6	0.65	3.64 (0.83)	1–6	0.57
Conflict	1.92 (0.66)	1–4	0.83	2.51 (0.87)	1–4	0.86
Inconsistent discipline	2.06 (0.70)	1–4	0.78	2.73 (0.86)	1–4	0.83

Note. *N* = 172–176 (Austria), *N* = 321–338 (Hungary); *ω* = McDonald’s *ω*.

**Table 2:** Correlations among HLEQ dimensions in Hungary (below diagonal) and Austria (above diagonal).

Variables	1	2	3	4	5	6	7	8	9	10
Child age (1)	-	−0.10	−0.09	−0.01	0.02	0.19 *	−0.16 *	0.05	0.00	0.03
Child gender (2)	0.05	-	0.05	0.02	−0.13	−0.04	0.01	−0.15	0.03	−0.00
Family Education (3)	−0.04	−0.01	-	−0.06	−0.10	−0.23 **	0.01	0.28 **	0.02	−0.10
Family Language (4)	0.11	−0.06	0.00	-	−0.18 *	−0.12	−0.02	−0.08	−0.17 *	−0.10
HLEQ IA (5)	−0.03	−0.13 *	0.11 *	0.08	-	0.27 **	0.48 **	0.41 **	0.14	0.12
HLEQ DA (6)	0.18 **	0.02	−0.22 **	0.14 *	−0.05	-	0.17 *	0.17 *	0.32 **	0.38 **
HLEQ WS (7)	−0.07	−0.03	0.07	0.11 *	0.46 **	0.12 *	-	0.28 **	−0.09	0.10
HLEQ OA (8)	0.12 *	−0.08	0.21 **	0.11 *	0.42 **	−0.05	0.24 **	-	0.17 *	0.01
HLEQ CO (9)	0.12 *	−0.01	0.06	−0.04	−0.30 **	0.10	−0.33 **	−0.16 **	-	0.64 **
HLEQ ID (10)	0.09	−0.08	0.00	−0.07	−0.16 **-	0.10	−0.24 **	−0.11 *	0.43 **	-

Note. Hungary below diagonal, Austria above diagonal. * *p* < 0.05, ** *p* < 0.01; *N* = 175–176 (Austria), *N* = 326–338 (Hungary). IA = Indoor learning activities, DA = Digital learning activities, WS = Warmth/Support, OA = Outdoor learning activities, CO = Conflict, ID = Inconsistent discipline.

**Table 3:** Adjusted means and ANCOVA results for country differences across HLEQ dimensions.

HLEQ Dimensions	Austria *M*_adj_	Hungary *M*_adj_	*M*_diff_ (AT–HUN)	*F*(1, 486)	*p*	*η* ^2^_p_
Indoor learning activities	5.12	5.21	−0.08	1.27	0.261	0.003
Digital learning activities	4.38	3.94	0.44	27.74	<0.001	0.054
Warmth/Support	2.17	1.68	0.49	37.59	<0.001	0.072
Outdoor learning activities	3.49	3.15	0.35	13.51	<0.001	0.027
Conflict	2.32	1.67	0.65	61.76	<0.001	0.113
Inconsistent discipline	2.65	1.87	0.78	84.84	<0.001	0.149

Note. Values represent estimated marginal means controlling for child age in months, maternal education, paternal education, gender, and family language. η^2^_p_ = partial eta squared.

**Table 4:** Measurement invariance of the HLEQ model across Hungarian and Austrian samples.

Model	χ^2^	df	CFI	RMSEA	SRMR	ΔCFI
Configural	1328.14	886	0.905	0.047	0.064	-
Metric	1375.13	912	0.901	0.048	0.069	0.004
Scalar	1703.03	938	0.836	0.061	0.078	0.065
Partial scalar	1572.14	934	0.863	0.056	0.074	0.038

Note. Robust fit indices are reported because models were estimated using robust maximum likelihood (MLR). ΔCFI values were calculated relative to the less constrained preceding model.

## Data Availability

The complete anonymized dataset and further research material are available at https://osf.io/7syaz (accessed on 16 June 2026).

## Supplementary Materials

The following supporting information can be downloaded at: https://www.mdpi.com/article/10.3390/bs16071111/s1, Table S1: Sample Characteristics of Austrian and Hungarian Participants; Table S2: Residual correlations included in the refined HLEQ CFA model; Table S3: Standardized factor loadings of the HLEQ items in Hungary and Austria; Table S4: Freed intercepts in the exploratory partial scalar invariance model.
