# Supplementary material for: Exploring Children’s Digital Home Learning Environment: Cross-Cultural Construct Validation of the HLEQ
Source: Behav Sci (Basel). 2026 Jul 3;16(7):1111. doi: 10.3390/bs16071111 (PMC13405998; doi:10.3390/bs16071111)
Supplement: Supplementary file 1 [file behavsci-16-01111-s001.zip › behavsci-4306102-supplementary.pdf]

Supplement Table S1

Sample Characteristics of Austrian and Hungarian Participants

Variable	Hungary (N = 339)		Austria (N = 176)	
	N	Valid percent (%)	N	Valid percent (%)
Gender:				
Male	162	47.5	85	48.3
Female	177	52.5	91	51.7
Mother education:				
No formal education	7	2.1	2	1.1
Middle school	32	9.5	0	0
Technical school/ vocational training	126	37.5	16	9.1
High school diploma	80	23.8	33	18.8
University diploma	91	27.1	125	71.0
Father education:				
No formal education	7	2.1	1	.6
Middle school	79	23.7	6	3.4
Technical school/ vocational training	105	31.5	36	20.5
High school diploma	61	18.3	38	21.6
University diploma	81	24.3	95	54.0
Language:				
Only Hungarian/German	302	92.1	130	74.3
Mostly Hungarian/German	26	7.9	21	12.0
Mainly other language than Hungarian/German	0	0	12	6.9
Other language and Hungarian/German as often	0	0	12	6.9
Mean age in month	63.73		57.64	
SD	12.54		13.12	

Supplement Table S2

Residual correlations included in the refined HLEQ CFA model

Item pair
HLEQ_OA_3 ~~ HLEQ_OA_4
HLEQ_OA_2 ~~ HLEQ_OA_4
HLEQ_DA_2 ~~ HLEQ_DA_3
HLEQ_WS_2 ~~ HLEQ_WS_3
HLEQ_WS_1 ~~ HLEQ_WS_4
HLEQ_CO_1 ~~ HLEQ_CO_2

Note. *Residual covariances were added only within subscales and were retained based on theoretical plausibility and empirical model improvement.*

Supplement Table S3

Standardized factor loadings of the HLEQ items in Hungary and Austria

Factor	Item	Hungary (λ)	Austria (λ)
IA	HLEQ_IA_1	.47	.43
IA	HLEQ_IA_2	.35	.41
IA	HLEQ_IA_3	.69	.49
IA	HLEQ_IA_4	.71	.63
IA	HLEQ_IA_5	.63	.54
IA	HLEQ_IA_6	.63	.45
DA	HLEQ_DA_1	.43	.42
DA	HLEQ_DA_2	.49	.63
DA	HLEQ_DA_3	.55	.61
DA	HLEQ_DA_4	.63	.53
DA	HLEQ_DA_5	.54	.73
WS	HLEQ_WS_1	.74	.66
WS	HLEQ_WS_2	.70	.72
WS	HLEQ_WS_3	.70	.77
WS	HLEQ_WS_4	.79	.71
WS	HLEQ_WS_5	.59	.53
WS	HLEQ_WS_6	.64	.45
OA	HLEQ_OA_1	.32	.54
OA	HLEQ_OA_2	.69	.45
OA	HLEQ_OA_3	.33	.43
OA	HLEQ_OA_4	.56	.47
OA	HLEQ_OA_5	.54	.39
CO	HLEQ_CO_1	.70	.61
CO	HLEQ_CO_2	.60	.73
CO	HLEQ_CO_3	.78	.82

Factor	Item	Hungary (λ)	Austria (λ)
CO	HLEQ_CO_4	.64	.73
CO	HLEQ_CO_5	.78	.83
ID	HLEQ_ID_1	.66	.71
ID	HLEQ_ID_2	.83	.76
ID	HLEQ_ID_3	.77	.75
ID	HLEQ_ID_4	.45	.74
ID	HLEQ_ID_5	.55	.52

Note. *All factor loadings were statistically significant ($p < .001$).*

Supplement table S4

Freed intercepts in the exploratory partial scalar invariance model

**Item**	**Freed parameter**
HLEQ_IA_1	Intercept
HLEQ_DA_3	Intercept
HLEQ_OA_1	Intercept
HLEQ_ID_1	Intercept

Note. *These item intercepts were freely estimated across Hungary and Austria in an exploratory attempt to improve scalar model fit.*
